# Critical contributions of neuronal subtypes to pediatric drug-resistant focal dysplasia

**DOI:** 10.3389/fcell.2025.1566137

**Published:** 2025-04-17

**Authors:** Yujie Zhang, Lin Li, Fengjun Zhu, Xinyang Zhang, Dan Xia, Qiuling Miao, Cheng Zhong, Shuli Liang, Dezhi Cao, Huafang Zou, Jing Duan, Yousheng Shu, Yi Yao, Jianming Song, Songnian Hu, Jianxiang Liao, Qiang Zhou

**Affiliations:** ^1^ Pediatric Neurology, Shenzhen Children’s Hospital, Shenzhen, China; ^2^ Surgery Division, Epilepsy Center, Shenzhen Children’s Hospital, Shenzhen, Guangdong, China; ^3^ State Key Laboratory of Chemical Oncogenomics, Shenzhen Key Laboratory of Chemical Genomics, Peking University Shenzhen Graduate School, Shenzhen, China; ^4^ Cellular and Molecular Diagnostics Center, Sun Yat-sen Memorial Hospital, Sun Yat-sen University, Guangzhou, China; ^5^ Department of Pathology, Shenzhen Children’s Hospital, Shenzhen, Guangdong, China; ^6^ State Key Laboratory of Brain Cognition and Brain-inspired Intelligence Technology, Shenzhen Institutes of Advanced Technology, Chinese Academy of Sciences, Shenzhen, Guangdong, China; ^7^ Shenzhen Key Laboratory of Precision Diagnosis and Treatment of Depression, CAS Key Laboratory of Brain Connectome and Manipulation, Shenzhen Institute of Advanced Technology, Chinese Academy of Sciences, Shenzhen, Guangdong, China; ^8^ Functional Neurosurgery Department, National Children’s Health Center of China, Beijing Children’s Hospital, Capital Medical University, Beijing, China; ^9^ State Key Laboratory of Medical Neurobiology, Institute for Translational Brain Research, MOE Frontier Center for Brain Science, Department of Neurology, Huashan Hospital, Fudan University, Shanghai, China; ^10^ State Key Laboratory of Microbial Resources, Institute of Microbiology, Chinese Academy of Sciences, Beijing, China

**Keywords:** focal cortical dysplasia, drug-resistant epilepsy, neuronal subtypes, energy metabolism, fast-spiking neuron

## Abstract

Approximately 75% of epilepsy cases emerge in childhood, and 10%–30% of these pediatric epilepsy cases are resistant to standard drug therapies; however, the underlying causes of resistance remain poorly understood. Focal cortical dysplasia (FCD) is a primary contributor to pediatric epilepsy and is often associated with drug resistance. We performed single-nucleus RNA sequencing (snRNA-seq) and patch-clamp recording of fresh brain tissue samples that were obtained from pediatric FCD patients during surgery. Our study revealed significant transcriptomic changes across multiple subtypes of excitatory neurons and GABAergic neurons. Among the identified neuronal subtypes, the three inhibitory neuronal subtypes PVALB_RGS5, VIP_CRH, and SST_PENK presented prominent transcriptomic alterations related to epilepsy. The expression of genes enriched in epilepsy-related signaling pathways, especially those associated with excitatory/inhibitory (E/I) balance and energy metabolism, was significantly altered in these neuronal subtypes. Differentially expressed genes (DEGs) in the PVALB_RGS5 subtype were particularly enriched in pathways related to synaptic function. Recordings from fast-spiking (FS)/parvalbumin-containing neurons in brain sections from patients with FCD revealed a reduction in excitatory synaptic inputs, which indicates fewer synaptic inputs onto these neurons and lower activity. In addition, astrocyte subtype 4 exhibited distinct metabolic characteristics and interaction patterns with neuronal subtypes, which suggests their significant role in epilepsy pathophysiology. Our findings indicate that several specific neuronal and astrocyte subtypes play critical roles in the genesis and/or progression of drug-resistant pediatric seizures and that targeting these subtypes may represent a new treatment option.

## Introduction

The prevalence (ranging from 3.2 to 5.5/1,000 in developed countries and 3.6–44/1,000 in underdeveloped countries) and incidence (41–187/100,000) of epilepsy in children are high, and approximately 75% of epilepsy cases begin in childhood (Stafstrom and Carmant, 2015; Camfield and Camfield, 2015). Approximately 10%–30% of children with epilepsy have drug-resistant epilepsy, which cannot be fully controlled by medications (Wu et al., 2020). Since poorly controlled seizures severely affect the development and health of pediatric epilepsy patients, better treatments based on an understanding of the cellular and molecular alterations in the pediatric population are critical.

Focal cortical dysplasia (FCD) is a type of malformation of cortical development (MCD) that is characterized by disrupted cytoarchitecture of the cerebral cortex (Balestrini et al., 2023). FCD is strongly associated with drug-resistant epilepsy and is the most common etiology in children with drug-resistant focal epilepsies (Guerrini and Barba, 2021). More than 50% of FCD cases are associated with temporal lobe epilepsy (TLE) (Wu et al., 2014). Approximately 61% of FCD patients experience seizure onset before age 5, and 92.5% experience seizure onset before age 16 (Fauser et al., 2006). The pathophysiology of FCD and its association with epilepsy remain poorly understood.

Both excitatory and inhibitory neurons can be divided into numerous subtypes. A single functional area of the human cortex contains 45 inhibitory and 24 excitatory neuron subtypes (Hodge et al., 2019), whereas 13 excitatory and 23 inhibitory neuron subtypes have been identified in adult TLE patients, with only a few subtypes displaying significant transcriptomic alterations (Pfisterer et al., 2020). These findings suggest that epilepsy is associated with or caused by pathological alterations in only a few specific neuronal subtypes. Studying these neuronal subtypes and identifying the genes most significantly affected may provide crucial insight into the genesis and treatment of epilepsy. However, whether few neuronal subtypes are selectively altered in pediatric FCD is unknown.

Aberrant signaling from astrocytes to neurons significantly contributes to hyperexcitability in neural networks (Nikolic et al., 2020). Dysregulation of astrocyte functions, including gliotransmission, cellular metabolism, and the immune response, significantly contribute to the emergence and progression of epileptic hyperexcitability (Vezzani et al., 2022). Astrocytes also play a key role in supplying energy to neurons, and impaired energy homeostasis in astrocytes contributes to epilepsy pathogenesis (Zhang Y. M. et al., 2023). A few selective astrocyte subtypes have been shown to significantly influence epilepsy (Qian et al., 2023; Chung et al., 2023). However, the specific contributions of astrocytes to pediatric epilepsy, including FCD, are unknown.

To address these questions regarding pediatric epilepsy, we performed single-nucleus RNA sequencing (snRNA-seq), which is an effective and accurate method for identifying cell subtypes. Previously, snRNA-seq revealed selective transcriptomic alterations in specific neuronal subtypes in the brains of adults with epilepsy (Pfisterer et al., 2020) and selective enrichment of MCD-related gene sets in astrocyte subtypes and oligodendrocytes from pediatrics MCD patients (Chung et al., 2023). Although postmortem tissue samples are typically used for snRNA-seq, the use of fresh tissues is better for revealing dynamic changes in the brain (Dachet et al., 2021; Heng et al., 2021). When postmortem tissue was compared with fresh human brain tissue, a decrease in the level of neuronal activity-dependent transcripts was observed (Dachet et al., 2021). Thus, the use of fresh brain tissue samples is especially important when analyzing the expression of genes that are affected by deteriorated cell health, such as mitochondrial genes.

In this study, we performed snRNA-seq of fresh brain tissues from pediatric patients with drug-resistant FCD. Three inhibitory neuronal subtypes, PVALB_RGS5, VIP_CRH, and SST_PENK, presented significant transcriptomic alterations related to epilepsy. Astrocyte subtype 4 also exhibited unique metabolic features and interactions with neuronal subtypes and may play a critical role in epilepsy pathophysiology.

## Materials and methods

### Sample collection

Human brain samples were collected from temporal lobectomies of pediatric patients undergoing surgery for FCD treatment at Shenzhen Children’s Hospital. The detailed clinical information of the patients is presented in Supplemenatary Table S1. All participants were enrolled from April 2021 to October 2023. This study was approved by the Institutional Ethics Committee of Shenzhen Children’s Hospital (ethics approval number: 202,002,302), and all enrolled patients and their guardians provided informed consent.

### Sample preparation

Precooled PBS containing 2 mM EGTA (PBSE) was used to wash the brain tissue. The nuclei were then isolated with GEXSCOPE® Nucleus Separation Solution (Singleron Biotechnologies, Nanjing, China) according to the product manual. The isolated nuclei were resuspended in PBSE at a concentration of 10^6^ nuclei per 400 μL, filtered with a 40 μm cell strainer, and counted with Trypan blue. The nuclei in PBSE were stained with DAPI (1:1,000) (Thermo Fisher Scientific, D1306), and nuclei were defined as DAPI-positive singlets.

### Primary analysis of raw read data

Gene expression matrixes were generated from raw snRNA-seq reads with the CeleScope v1.9.0 pipeline (https://github.com/singleron-RD/CeleScope). In brief, to remove low-quality reads, raw reads were first processed using CeleScope with Cutadapt v1.17, after which the poly-A tail and adapter sequences were trimmed (Martin, 2011). After the cell barcodes and UMI were extracted, STAR v2.6.1a (Dobin et al., 2013) was used to map the reads to the reference genome GRCh38 (ensembl version 92 annotation). We subsequently acquired UMI counts and gene counts for each cell with featureCounts v2.0.1 software (Liao et al., 2014). The UMI counts and gene counts for each cell were used to generate expression matrix files for subsequent analysis.

### snRNA-seq library preparation

The concentration of single-nucleus suspensions was adjusted to 3–4 × 10^5^ nuclei/mL with PBS. The single-nucleus suspensions were loaded onto microfluidic chips (GEXSCOPE® Single Nucleus RNA-seq Kit, Singleron Biotechnologies), and snRNA-seq libraries were constructed according to the manufacturer’s instructions (Singleron Biotechnologies). An Illumina Nova seq 6,000 instrument was used to sequence the snRNA-seq libraries with 150 bp paired-end reads.

### Quality control, dimensionality reduction and clustering

Scanpy v1.8.2 was used for quality control (Wolf et al., 2018), dimensionality reduction and clustering with Python 3.7. For each sample, cells were excluded if they met the following criteria: (1) cells with a gene count less than 200 or with a top 2% gene count; (2) cells with a top 2% UMI count; (3) cells with a mitochondrial content >20%; and (4) genes expressed in fewer than five cells.

We obtained 102,974 cells for the downstream analyses, with an average of 2098 genes and 4,537 UMIs per cell. The raw count matrix was normalized to the total count per cell and logarithmically transformed into a normalized data matrix. The top 2,000 variable genes were selected by setting the flavor to ‘seurat’. Principal component analysis (PCA) of the scaled variable gene matrix was performed, and the top 20 principal components were used for clustering and dimensionality reduction. The uniform manifold approximation and projection (UMAP) algorithm was applied to visualize cells in a two-dimensional space.

### Differentially expressed gene (DEG) analysis

To identify DEGs, the scanpy. tl.rank_genes_groups () function based on the Wilcoxon rank sum test with default parameters was used. Genes expressed in more than 10% of the cells in at least one of the two groups and with a |fold change| ≥2 were considered differentially expressed. The adjusted p value (*p.adjust*) was calculated using the Benjamini‒Hochberg correction. *P. adjust* < 0.05 was used as the criterion for statistical significance.

### Cell type annotation

The identity of each cell cluster was determined based on the expression of canonical markers found in the DEGs using the SynEcoSys database. Heatmaps/dot plots displaying the expression of the markers used to identify each cell type were generated via Seurat v3.1.2 DoHeatmap/DotPlot/Vlnplot.

### Pathway enrichment analysis

To investigate the potential functions of the DEGs, Gene Ontology (GO) and Kyoto Encyclopedia of Genes and Genomes (KEGG) analyses were performed using the “clusterProfiler” R package 3.16.1 (Yu et al., 2012). Pathways with *p. adjust* values <0.05 were considered significantly enriched. GO gene sets, including the molecular function (MF), biological process (BP), and cellular component (CC) categories, were used as references.

To investigate the expression of some pathway-related genes in different neuronal subtypes, pathway-related genes were identified and used to create functional gene sets for U Cell gene set scoring. The query genes were ranked in order of their expression levels in individual cells, and U Cell scores were determined by the Mann‒Whitney U test (Andreatta and Carmona, 2021). The U Cell score ratio was calculated as (Ep - Ctrl) * 1,000/Ctrl.

### Cell‒cell interaction analysis

CellChat (version 0.0.2) was used to analyze intercellular communication networks from the scRNA-seq data (Jin et al., 2021). A CellChat object was created using the R package. First, the gene expression data were projected onto a protein‒protein interaction network. Then, the computeCommunProbPathway and aggregateNet functions were utilized to assign probability values to predict the intercellular communication network. Centrality indices of components of the interaction network were subsequently calculated to elucidate the role and plasticity of each cell population in various signaling pathways. The netVisual_circle function was employed for visualization of the intercellular communication network and analysis of network strength. Finally, visualization of intercellular communication and the intensity of ligand‒receptor interactions between different cell populations was evaluated using the netVisual_bubble function on the basis of a database of human ligand‒receptor pairs.

### Hematoxylin‒eosin (HE) staining

To assess pathological changes in the sequenced tissue, the following procedure was performed. First, the tissues were sectioned to a thickness of 1 mm. Each slice was then numbered according to their order. The odd-numbered sections were placed in 10% polyformaldehyde, while the even-numbered sections were placed in cryotubes and frozen at −80°C in liquid nitrogen. Then, the sections were fixed in 10% polyformaldehyde (pH 7.4), washed, dehydrated, made transparent, and embedded in paraffin into wax blocks. The paraffin blocks were sliced into 4 µm thick sections. These sections were then stained with hematoxylin and eosin, and cell morphology was examined under a light microscope (Olympus APX100, Japan). If clear features of FCD1a (such as cells with a distinct columnar arrangement) were observed in the consecutively numbered sections, the section between the stained sections (which were frozen in liquid nitrogen) was included in the epilepsy group for RNA-seq analysis. Using the same method, the suitability of the tissue for inclusion in the control group was determined on the basis of HE staining results.

### Whole-cell recording

Human brain tissues were rapidly removed and placed in ice-cold sucrose-ACSF consisting of (in mM): 126 sucrose, 2 MgSO_4_, 2.5 KCl, 1.25 NaH_2_PO_4_, 26 NaHCO_3_, 10 D-glucose, and 2 CaCl_2_ (gassed with 95% O_2_ and 5% CO_2_). Slices (350 μm thick) were cut with a Leica VT1200 tissue slicer (Germany) in ice-cold sucrose-ACSF. The slices were transferred to a holding chamber with normal ACSF containing (in mM): 126 NaCl, 2.5 KCl, 1.25 NaH_2_PO_4_, 26 NaHCO_3_, 25 D-glucose, 2 CaCl_2_, and 2 MgSO_4_. After sectioning, the slices were allowed to recover for 40 min at 34°C and then kept at room temperature until recording. During recording, the slices were perfused with 35°C–36°C oxygenated modified ACSF at 4–5 mL/min (modified from normal ACSF; containing (in mM) 126 NaCl, 3.5 KCl, 1.25 NaH_2_PO_4_, 26 NaHCO_3_, 25 D-glucose, 1 CaCl_2_, and 1 MgSO_4_). Recordings were conducted under an Olympus microscope (BX51WI) with a 40 × water-immersion differential interference contrast objective. The resistance of the recording pipette was 4–8 MΩ. The recording pipettes were filled with K^+^ gluconate-based intracellular solution containing (in mM) 128 K^+^-gluconate, 10 NaCl, 2 MgCl_2_, 10 HEPES, 0.5 EGTA, 4 Na_2_ATP, and 0.4 NaGTP.

Recordings were obtained for fast-spiking (FS) neurons on the slice surface. A HEKA EPC10 double patch clamp amplifier was used. The signals were collected at a sampling rate of 10 kHz and filtered at 2 kHz. Neurons with a holding current > −200 pA (at - 70 mV) were excluded from the data analysis. To record spontaneous excitatory postsynaptic currents (sEPSCs), somatic whole-cell voltage clamp recordings (at −70 mV) were obtained. All neurons were recorded for at least 5 min to collect sEPSCs. Whole-cell current clamp recording of evoked spikes was performed using a series of 500 ms depolarizing current steps with an increment of 20 pA (from 0 to 200 pA) at 4 s intervals.

We used a few parameters to examine action potential (AP) properties. The first AP evoked by the rheobase was used to characterize the AP shape. The AP threshold was determined by the third derivative of the AP over the rising phase. The AP amplitude was calculated as the maximum amplitude from the AP peak to −70 mV (holding Vm). Afterhyperpolarization (AHP) was measured using the AHP peak and latency. The AHP peak was the difference in membrane potential between the AP threshold and trough (lowest point of the AP), whereas the AHP latency was the time difference between the AP peak and trough. More than 400 sEPSC events were identified for each neuron and analyzed using Mini Analysis software. The mean amplitude of sEPSCs for each neuron was used for group analysis.

FS neurons were infused with calcein (4 mmol/L). The spiking pattern was used to identify FS neurons, and sEPSCs were recorded at −70 mV. The recording pipettes were slowly removed, and a microscope was used to determine that the cells had been infused with green dye and remained on the brain slice. To confirm that the recorded neurons were PV positive, we performed immunofluorescence staining. The slices were fixed with 4% paraformaldehyde for 24 h and then dehydrated in 30% sucrose for 48 h at 4°C. The sections were washed with PBS three times for 10 min each and blocked with 10% normal goat serum containing 0.5% Triton-100 in PBS for 1 h at room temperature. The sections were incubated with a rabbit anti-parvalbumin antibody (1:2000, Abcam) at 4°C overnight, washed three times in PBS and incubated with an Alexa Fluor 546-conjugated secondary antibody (goat anti-rabbit 546; 1:400; Invitrogen) for 1 h at room temperature. The sections were then washed in PBS, cover-slipped, and imaged with a confocal microscope (Nikon A1R).

### Statistical analysis

The unpaired two-tailed Wilcoxon rank sum test was used for comparisons of gene expression or gene signatures between two groups. All the statistical analyses were performed using compare_means () in R. *P* < 0.05 was considered statistically significant.

## Results

### Distinct neuronal subtypes in brain tissues from pediatric FCD patients

**FIGURE 1:**
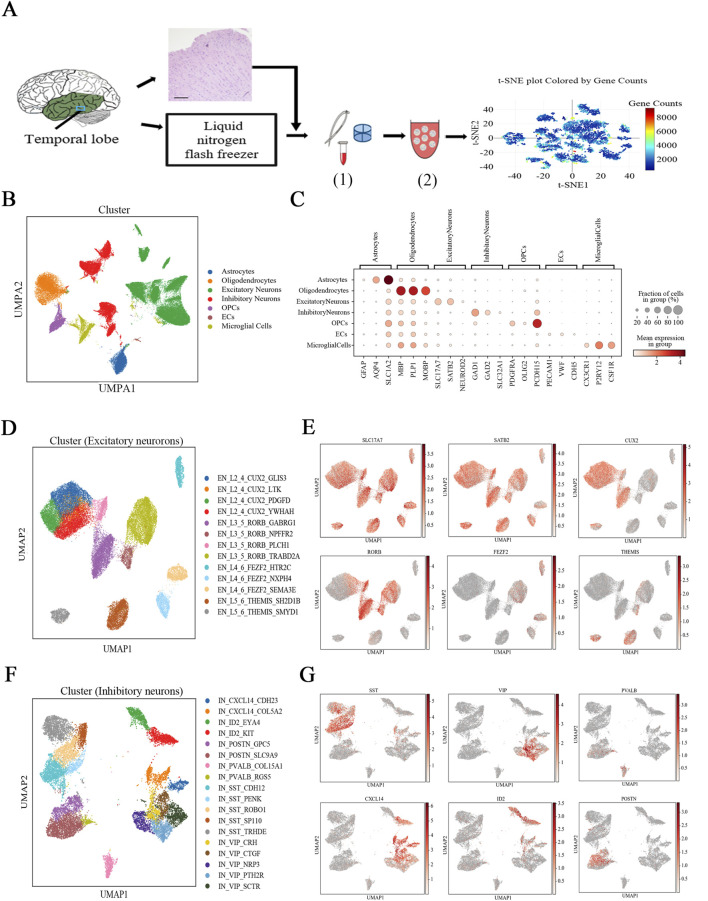
Overview of the single-nucleus transcriptomic dataset. **(A)** Experimental procedure for snRNA-seq analysis of brain tissues from pediatric FCD patients. (1) Tissue dissociation and digestion. (2) Library construction. Scale bar: 200 µm. **(B)** UMAP representation of the snRNA-seq data and cell type annotations based on the expression of known marker genes. **(C)** Expression of known marker genes for all cell types. **(D)** UMAP embedding and cell type annotation visualized for excitatory neuron subtypes. **(E)** Expression of general and family-specific marker genes in excitatory neurons. **(F)** UMAP embedding and cell type annotation for inhibitory neuron subtypes. **(G)** Expression of general and family-specific marker genes in inhibitory neurons. In **(E, G)**, the shade of red is proportional to the log of the normalized expression value.

Temporal lobe tissue samples from pediatric FCD patients were obtained during surgery and analyzed via snRNA-seq (Figure 1A). The epileptic focus zone was identified before surgery, and the temporal lobe was identified as the focus lesion center on the basis of magnetic resonance imaging (MRI), electroencephalogram (EEG) recordings, and positron emission tomography (PET) scans. Given the limitations of postmortem tissue and difficulty in obtaining pediatric postmortem tissue, we selected control section from fresh brain tissue samples obtained from regions distal to the epileptic focus zone, which were resected during surgery in patients with drug resistant seizures (Darmanis et al., 2015; Liu et al., 2024; Calcagnotto et al., 2005). From these tissues, we selected sections with no apparent histological aberrations as controls. Importantly, these tissues were not resected specifically for research purposes but rather as part of the necessary surgical treatment for optimal control of seizure.

HE staining was performed on the brain tissue samples to identify pathological alterations (Figure 1A). For tissue analysis, we sectioned the epilepsy zone into 1 mm slices and numbered each slice consecutively. Odd-numbered sections were fixed in 10% polyformaldehyde for HE staining, while even-numbered sections were frozen in liquid nitrogen at −80°C for RNA-seq analysis (see Methods for details). If HE staining revealed a visible columnar organization of neurons (indicative of FCD1a) in the odd-numbered sections (Supplementary Figure S1A), the adjacent even-numbered frozen section was assigned to the epilepsy group. Conversely, if no columnar organization was observed, the adjacent frozen section was assigned to the control group (Supplementary Figure S1B).

The epilepsy group contained eight samples from eight patients, and the control group contained six samples from six patients (Supplementary Figure S2A, B), which were matched for age and median gene expression (Supplemenatary Table S1; Supplementary Figure S2C, D). A total of 85,716 single-nuclei gene expression profiles passed quality control and were used for further analysis (Supplemenatary Table S2).

By using snRNA-seq, we identified seven distinct cell types on the basis of the expression of known cell type markers (Supplemenatary Table S3; Figures 1B, C), and there was a total of 39,861 excitatory and 19,529 inhibitory neurons. The excitatory neurons were divided into 13 subtypes (Figure 1D), and the inhibitory neurons were divided into 18 subtypes (Figure 1F). Excitatory neurons were classified on the basis of the expression of previously described layer-specific marker genes *CUX2* (L2_4), *RORB* (L3_5), *FEZF2* (L4_6), and *THEMIS* (L5_6) (Figure 1E), and inhibitory neurons were classified on the basis of the expression of the key marker genes *PVALB*, *SST*, *VIP*, *CXCL14*, *POSTN*, and *ID2* (Figure 1G) (Pfisterer et al., 2020). A third hierarchical level (for example, *YWHAH* or *LTK*) was created by comparing DEGs with the highest expression in a given subtype to DEGs in the other subtypes (Figures 1E, G; Supplemenatary Table S3). We thus identified 13 excitatory neuronal subtypes and 18 inhibitory neuronal subtypes. There was no significant difference in the percentage of cells of each neuronal subtype relative to the entire neuronal population between the epilepsy and control groups (Supplementary Figure S2E, F).

### Prominent epilepsy-related alterations in select neuronal subtypes

**FIGURE 2:**
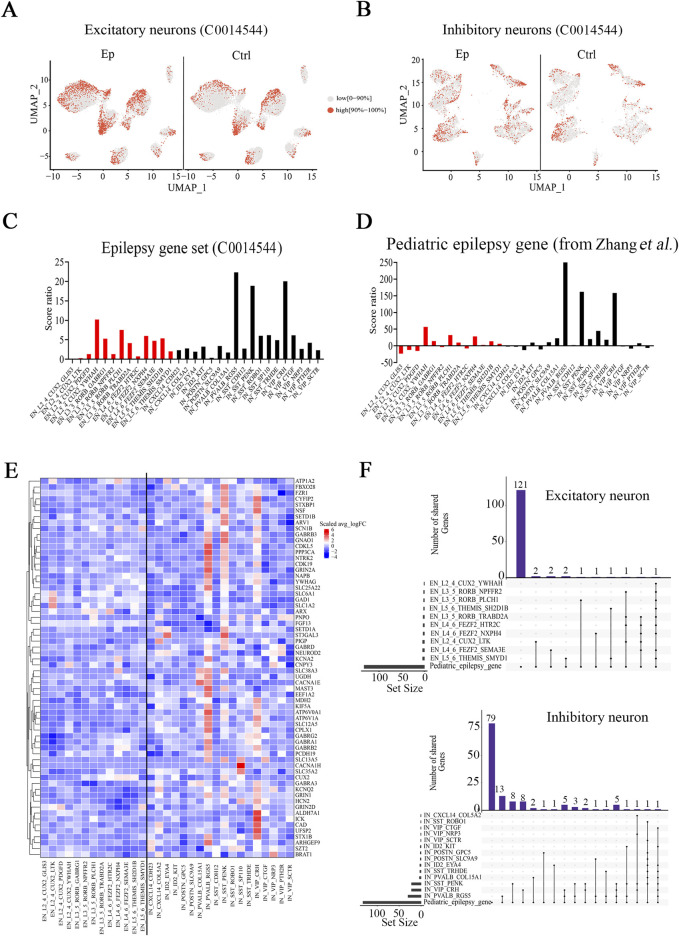
Epilepsy-related neuronal subtypes in the brain tissues of pediatric FCD patients. **(A)** UMAP representation of the scores for epilepsy genes identified with DisGeNET (C0014544) in excitatory neuronal subtypes in the epilepsy and control groups. **(B)** UMAP representation of the scores for epilepsy genes identified with DisGeNET (C0014544) in inhibitory neuronal subtypes in the epilepsy and control groups. **(C)** Score ratios for epilepsy genes identified with DisGeNET in excitatory and inhibitory neuronal subtypes. **(D)** Score ratios for the pediatric epilepsy gene set (from Zhang et al.) in excitatory and inhibitory neuronal subtypes. **(E)** Relative expression (epilepsy (Ep) vs control (Ctrl)) of pediatric epilepsy genes (from Zhang et al.) in excitatory and inhibitory neuronal subtypes. **(F)** UpSet plot showing the numbers of overlapping pediatric epilepsy genes and DEGs (Ep vs Ctrl) in identified neuronal subtypes. In **(A)** and **(B)**, red represents high scores (the top 10%), and gray represents low scores (0%–90%). In **(C)** and **(D)**, red represents the excitatory neuronal subtype, and black represents the inhibitory neuronal subtype. In **(E)**, the rows represent pediatric epilepsy genes, and the columns represent neuronal subtypes. The colors represent the scale (average log_2_ (fold change)). Fold change = G1/G2, where G1 and G2 represent gene expression in the epilepsy and control groups, respectively. In **(F)** On the bottom left hand side, the horizontal bar chart on the bottom left-hand side represents the entire size of each set. In the upper right-hand corner, the values on top of the bars indicate the numbers of shared genes at each intersection. Unconnected individual dots represent unique genes found in the neuronal subtypes. Dots connected by lines indicate genes shared between two or more neuronal subtypes, which correspond to the numbers on the bars in the upper section of the graph.

Our objective was to examine whether significant epilepsy-related alterations in gene expression occurred uniformly across the above-identified neuronal subtypes or whether only a few subtypes presented prominent alterations. First, we utilized the U Cell method to evaluate the expression of the epilepsy gene set (C0014544) and compared U Cell scores between the epilepsy and control groups. Scores were significantly greater for several neuronal subtypes in the epilepsy group (Figures 2A, B; Supplementary Figures S3A, B). The above conclusion was confirmed since several subtypes also exhibited higher scores in additional analysis using a different pediatric epilepsy gene database (Supplemenatary Table S4-1, from Zhang) (Zhang M. W. et al., 2023) (Supplementary Figure S3C). These findings suggest that epilepsy gene sets were upregulated in neuronal subtypes based on higher U-cell scores. We subsequently calculated the score ratio for both genes in the epilepsy gene set (C0014544) and those in the pediatric epilepsy gene set from Zhang (Zhang M. W. et al., 2023) (Figures 2C, D; Supplemenatary Table S4-2) to identify variations in the neuronal subtypes with greater precision. Among the excitatory subtypes, L2_4_CUX2_YWHAH, L3_5_RORB_PLCH1, and L4_6_FEZF2_NXPH4 presented higher score ratios than the other excitatory neuronal subtypes did. Among the inhibitory neuronal subtypes, PVALB_RGS5, VIP_CRH, and SST_PENK presented significantly higher score ratios than the other inhibitory subtypes or other excitatory subtypes did. Analysis of gene sets from Zhang (Zhang M. W. et al., 2023) and Macnee (Macnee et al., 2023) confirmed this finding (Supplementary Figure S3D; Supplemenatary Tables S4-1, S4-3, S4-4).

To further investigate the relevance of the DEGs (Ep vs. ctrl) to pediatric epilepsy, we examined the overlap between the DEGs (Ep vs. ctrl) and pediatric epilepsy genes using UpSet analysis (Conway et al., 2017). Among the excitatory neuronal subtypes, the overlapping genes were sparse; a single overlap was identified in the L2_4_CUX2_YWHAH subtype, two overlaps were identified in the L3_5_RORB_PLCH1 subtype, and three were identified in the L4_6_FEZF2_NXPH4 subtype (Figures 2E, F). In contrast, there were 54 overlapping genes in inhibitory neurons, which indicates a more substantial difference in the expression of pediatric epilepsy genes. UpSet analysis revealed numerous DEGs within the three inhibitory neuronal subtypes PVALB_RGS5, VIP_CRH, and SST_PENK, in which the number of overlaps exceeded 20 (Figure 2E, F; Supplementary Figure S4; Supplemenatary Table S4-5). These genes were enriched in pathways associated with the transporter complex, ion channel and GABA receptor complex pathways (Supplemenatary Table S4-6). Among them, *GRIN2A*, *SLC12A5*, *SLC25A22*, and *KCNA2* exhibited increased expression in the PVALB_RGS5 subtype; *GRIN2D*, *GABRG2*, and *SCN1B* exhibited increased expression in the VIP_CRH subtype; and *GABRB3* exhibited increased expression in the SST_PENK subtype. Taken together, our results suggest that a few neuronal subtypes, particularly the PVALB_RGS5, VIP_CRH, and SST_PENK subtypes, are closely associated with pediatric epilepsy.

### Altered expression of genes related to neuronal development in brain tissues from FCD patients

**FIGURE 3:**
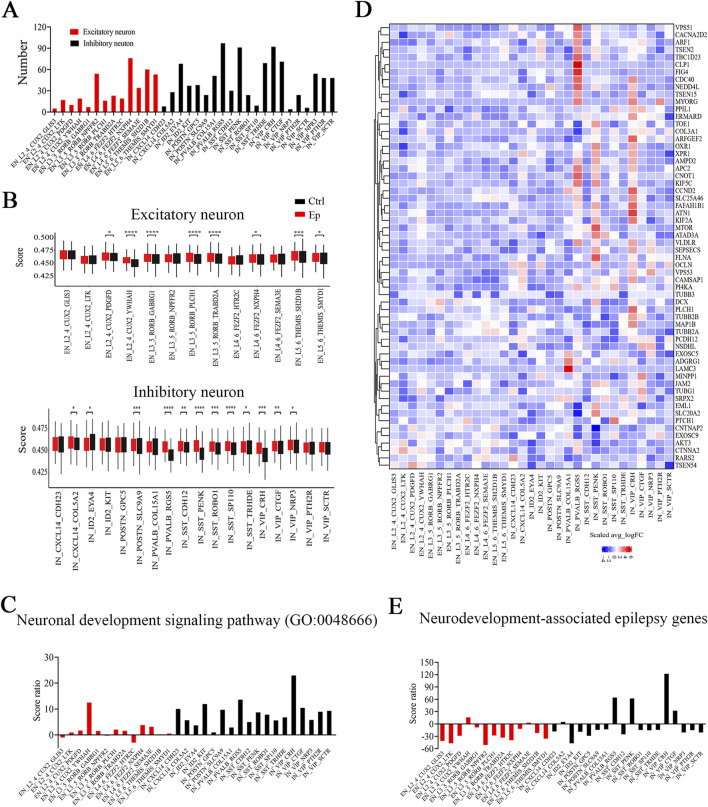
The expression of genes associated with neuronal development is altered in the brain tissues of pediatric FCD patients, especially in inhibitory neurons. **(A)** Numbers of enriched neuron function-related GO terms in neuronal subtypes. **(B)** Differences in U score of neuronal development signaling pathway (GO:0048666) in excitatory (top) and inhibitory (bottom) neuronal subtypes between the epilepsy and control groups. **(C)** Score ratios of neuronal development signaling pathway (GO:0048666) in neuronal subtypes. **(D)** Relative expression (Ep vs Ctrl) of neurodevelopment-associated epilepsy genes in excitatory or inhibitory neuronal subtypes. The rows represent neurodevelopment-associated epilepsy genes, and the columns represent neuronal subtypes. The colors represent the scale (avg_log_2_ (fold change)). Fold change = G1/G2, where G1 and G2 represent gene expression in the epilepsy and control groups, respectively. **(E)** Score ratios of neurodevelopment-associated epilepsy genes in excitatory and inhibitory neuronal subtypes. *, p < 0.05; **, p < 0.01; ***, p < 0.001; ****, p < 0.0001.

Most FCD patients experience the onset of symptoms during childhood, with approximately 92.5% of cases of FCD occurring before age 16 (Fauser et al., 2006). The age range of our patients was 5 months–15 years, which is consistent with the prominent role of neural development. GO enrichment analysis of the DEGs in each neuronal subtype revealed that the most strongly affected pathways were related to neuronal functions (Supplementary Figure S5). Among the neuronal subtypes, inhibitory neurons presented the greatest number of GO terms (>30) associated with neuronal functions, including synapse, synaptic transmission, and ion channel activity (Figure 3A; Supplementary Figure S5). This enrichment of function-related terms suggests that inhibitory neurons exhibit more alterations in epilepsy, and potentially reflects their critical role in maintaining network balance and their heightened susceptibility to dysregulation in disease. U-cell analysis revealed significantly higher scores for the neuronal development signaling pathway (GO:0048666) in most neuronal subtypes in the epilepsy group (Figure 3B). The VIP_CRH and L2_4_CUX2_YWHAH subtypes presented the highest score ratios for the neuronal development signaling pathway among the inhibitory and excitatory neuronal subtypes, respectively, which suggests that the most prominent alterations in this signaling pathway occurred in these two subtypes. In addition, most inhibitory neuronal subtypes presented higher score ratios compared to excitatory neuronal subtypes (Figure 3C; Supplemenatary Table S5). The score ratio, calculated as the difference in U-cell scores (reflecting pathway-related gene set activation) between epilepsy and control groups divided by the control group score, indicated greater activation of pathway-related genes in the inhibitory neurons. These findings suggest more pronounced functional alterations in the inhibitory neurons, which is consistent with their critical role in neural network balance and heightens their vulnerability in epilepsy. Conversely, some neuronal subtypes presented a decrease in the score ratio, suggesting a reduced activation or downregulation of the pathway-related gene sets. This may reflect functional suppression or compensatory processes in these neurons, which may potentially contribute to neural network stability in epilepsy.

Next, we focused on genes with mutations associated with both neurodevelopmental and focal or multifocal brain malformations (Zhang M. W. et al., 2023). In the epilepsy group, many genes were highly expressed in the three identified inhibitory neuronal subtypes (Figure 3D; Supplemenatary Table S6). Using U-cell analysis, we also observed that the three inhibitory neuronal subtypes and the VIP_CTGF subtype had higher score ratios than the other inhibitory neuronal subtypes did, and the L2_4_CUX2_YWHAH subtype had the highest score ratio among the excitatory neuronal subtypes (Figure 3E; Supplementary Figure S6; Supplemenatary Table S5). Overall, the three inhibitory neuronal subtypes and the L2_4_CUX2_YWHAH subtype presented pronounced increases in activity in both the neuronal development pathway and the neurodevelopmental focal or multifocal brain malformation gene set.

### Altered E/I balance-related signaling pathways and neuronal interactions in brain tissues from FCD patients

**FIGURE 4:**
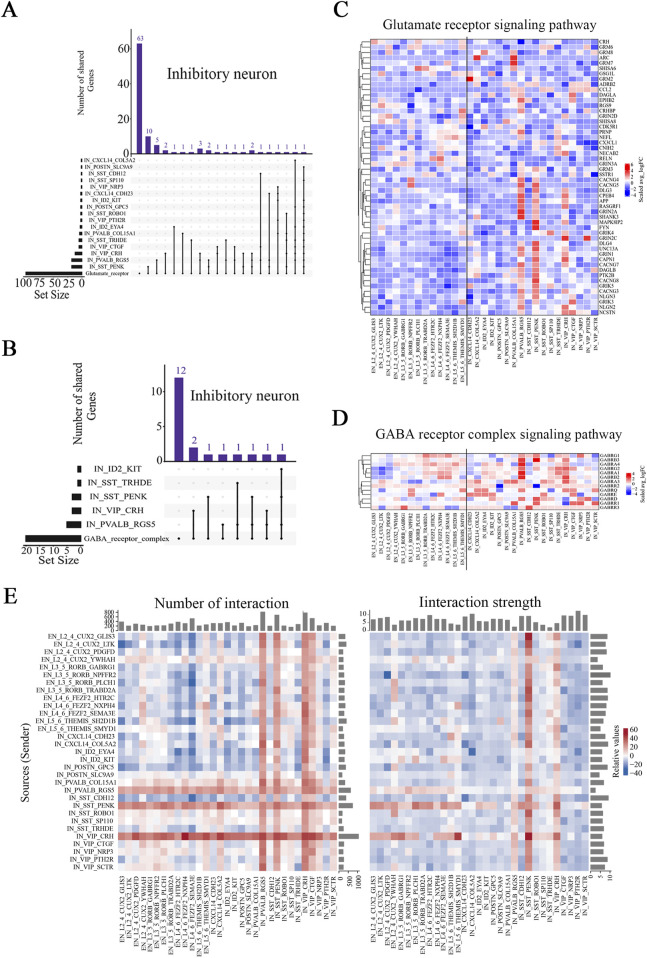
Major signaling pathways and neuronal interactions affected in the brain tissues of pediatric FCD patients. **(A)** UpSet plot showing the numbers of overlapping glutamate receptor signaling pathway-related genes and DEGs (Ep vs Ctrl) in the identified inhibitory neuronal subtypes. **(B)** UpSet plot showing the numbers of overlapping GABA receptor complex pathway-related genes and DEGs (Ep vs Ctrl) in the identified inhibitory neuronal subtypes. **(C)** Relative expression of genes (Ep vs Ctrl) in the glutamate receptor signaling pathway. **(D)** Relative expression of genes (Ep vs Ctrl) in the GABA receptor complex signaling pathway. **(E)** Differences in the number and strength of interaction pairs among distinct neuronal subtypes between the epilepsy and control groups. In **(A)** and **(B)**, the horizontal bar chart on the left-hand side represents the entire size of each set. In the upper right-hand corner, the values on top of the bars indicate the numbers of shared genes at each intersection. Unconnected individual dots represent unique genes found in the neuronal subtypes. Dots connected by lines indicate genes shared between two or more neuronal subtypes, which correspond to the numbers on the bars in the upper section of the graph. In **(C)** and **(D)**, the rows represent pathway-related genes, and the columns represent neuronal subtypes. The colors represent the scale (avg_log_2_ (fold change)). Fold change = G1/G2, where G1 and G2 represent gene expression in the epilepsy and control groups, respectively. In **(E)**, the rows represent receptor cell types, and the columns represent ligand cell types. The bar chart at the top indicates the sum of the column values shown in the incoming signals, whereas the bar chart on the right indicates the sum of the row values for outgoing signals. In the color bar, red (or blue) indicates an increase (or decrease) in the signal in the second dataset compared with the first dataset.

The excitatory/inhibitory (E/I) balance is often altered in epilepsy patients, which results in neuronal network hyperexcitability (Ziburkus et al., 2013). Glutamate and gamma-aminobutyric acid (GABA) are the predominant modulators of the E/I balance and epileptogenesis (Akyuz et al., 2021). We thus analyzed the expression of genes associated with the glutamate receptor signaling pathway (GO:0007215) and GABA receptor complex signaling pathways (GO:1902710). UpSet analysis revealed significant overlap between the DEGs in three inhibitory neuronal subtypes, PVALB_RGS5, VIP_CRH, and SST_PENK, and genes involved in the glutamate receptor and GABA receptor pathways (Figures 4A, B; Supplementary Figure S7). While excitatory neurons also contribute to these pathways, their DEGs in these pathways were less prominent (Supplementary Figure S7). This overlap was more pronounced in these three inhibitory neuronal subtypes than that in all other subtypes, suggesting that these three inhibitory neuronal subtypes were strongly associated with the E/I balance in the brains of FCD patients. Via U-cell analysis, we observed significantly higher scores for most subtypes in the epilepsy group (Supplementary Figures S8A, B). With respect to the glutamate receptor pathway, L2_4_CUX2_YWHAH and the three inhibitory neuronal subtypes presented higher score ratios than the other subtypes did, whereas with respect to the GABA receptor pathway, the VIP_CRH and PVALB_RGS5 subtypes presented higher score ratios (Supplemenatary Table S7; Supplementary Figures S8C, D).

Significant alterations in the glutamate receptor and GABA receptor signaling pathways may be used to identify genes involved in pediatric FCD, including both reported and unreported genes (Pfisterer et al., 2020). Among these*, GRIN2A, GABRB3*, and *GRIN2D* have been reported previously, supporting their roles in epilepsy pathophysiology (Chen et al., 2017; Strehlow et al., 2019; Absalom et al., 2022) (Figures 4C, D). In addition, no pathogenic variants exclusively associated with epilepsy or seizures have been identified for *CACNG7* and *CACNG8* (Mayo et al., 2023) (Figure 4C). Furthermore, we identified novel genes whose expression significantly changed in certain neuronal subtypes (Figures 4C, D; Supplemenatary Tables S8, S9). Selective and significant upregulation of *CRHBP*, *HOMER2*, and *GABRA4* was observed in the PVALB_RGS5 subtype; selective and significant upregulation of *MAPK8IP2*, *PTK2B*, *CACNG8*, and *GABRB3* was observed in the SST_PENK subtype; and selective and significant upregulation of *NECAB2*, *GABRA3*, and *GABRG2* was observed in the VIP_CRH subtype. Taken together, our findings reveal that DEGs in select subtypes are associated with E/I balance-related pathways, and we identified several previously unreported genes that exhibited significant expression changes in certain subtypes.

We also analyzed ligand-receptor-based interactions among neuronal subtypes using the CellChat R package. While a reduction in the number and strength of interaction pairs was noted across a broad spectrum of excitatory neuronal subtypes, the L2_4_CUX2_YWHAH subtype exhibited a relatively greater interaction probability than the other excitatory subtypes in the epilepsy group did, despite minimal changes in absolute interaction numbers and strength (Figure 4E; Supplementary Figures S8E, F). Among the inhibitory neurons, the number of interaction pairs tended to be greater in the epilepsy group than the control group, particularly for the PVALB_RGS5, VIP_CRH, and SST_PENK subtypes, and the VIP_CRH subtype showed the most significant increase (Figure 4E). Additionally, the VIP_CTGF subtype presented an increase in the number of interactions but a decrease in interaction strength, suggesting a potential shift in the quality or efficiency of its connections in epilepsy. Interestingly, while the strength of interactions was reduced in the majority of inhibitory neuronal subtypes, the aforementioned three subtypes exhibited stronger interactions with other neuronal subtypes in the epilepsy group (Supplementary Figure S8F). Collectively, our findings indicate complex alterations in the interactions among neuronal subtypes in epilepsy. Most notably, however, the interaction number and strength of the three inhibitory neuronal subtypes increased, which suggests that stronger interactions between these subtypes and other subtypes may play an important role in epilepsy.

### Altered ATP production and energy metabolism in the brain tissues of FCD patients

**FIGURE 5:**
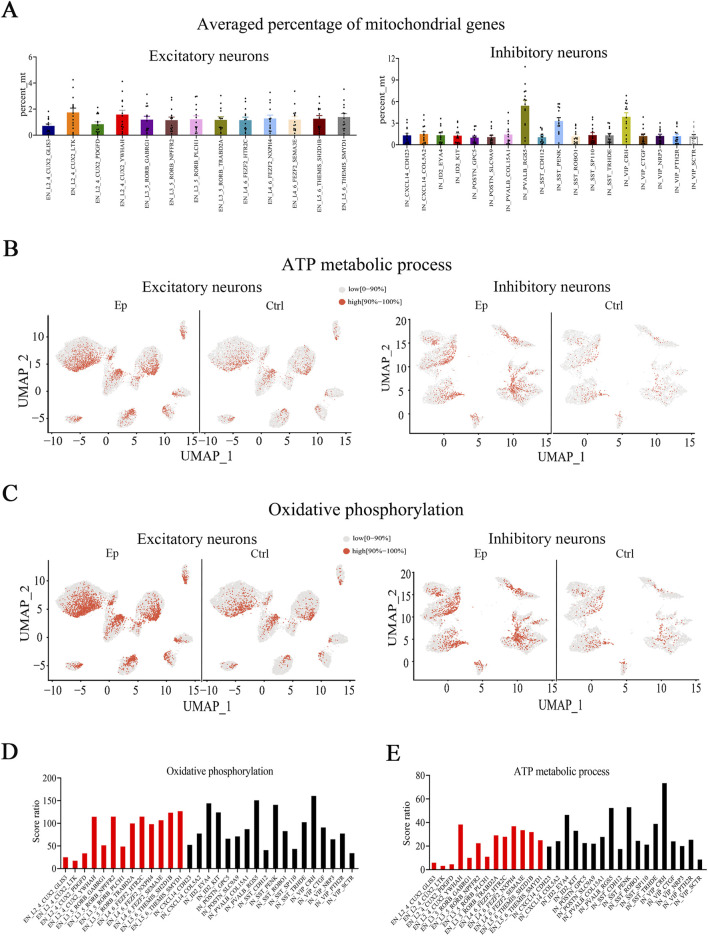
The expression of genes associated with mitochondrial functions are altered in the brain tissues of pediatric FCD patients. **(A)** Average percentage of mitochondrial genes in excitatory and inhibitory neuronal subtypes. **(B)** UMAP representation of the scores of genes involved in the ATP metabolic process pathway in excitatory and inhibitory neuronal subtypes in the epilepsy and control groups. **(C)** UMAP representation of the scores of genes involved in the oxidative phosphorylation pathway in excitatory and inhibitory neuronal subtypes in the epilepsy and control groups. **(D)** Score ratios for the oxidative phosphorylation signaling pathway in excitatory and inhibitory neuronal subtypes. **(E)** Score ratios for the ATP metabolic process signaling pathway in excitatory and inhibitory neuronal subtypes. For A, the vertical axis (percent_mt) represents the proportion of mitochondrial-encoded genes expressed within each neuronal subtype. In **(B)** and **(C)**, red represents high scores (the top 10%), and gray represents low scores (0%–90%).

Alterations in energy-related processes have been implicated in the development and/or maintenance of epilepsy, and impaired bioenergetics manifests as a breakdown of the mitochondrial machinery (Singh et al., 2021). However, in previous snRNA-seq analyses of epilepsy patients, postmortem tissues were used, which precluded the possibility of examining energy-related processes (Pfisterer et al., 2020). By using fresh tissue samples from FCD patients, we observed that DEGs (Ep vs ctrl) in most neuronal subtypes were enriched in pathways related to mitochondrial functions. A greater percentage of mitochondrial genes (the proportion of mitochondrial-encoded genes expressed in a given cell type or subtype) was found in the three inhibitory subtypes than in the other neuronal subtypes (Figure 5A). This difference was observed in most tissue samples (Supplementary Figures S9, S10). Importantly, this difference occurred in the epilepsy and control groups; hence, it reflects a unique neuronal feature of these subtypes but is likely not due to epilepsy. Mitochondria play a critical role in generating energy *via* ATP production, and we observed that the DEGs in many subtypes were enriched in both the oxidative phosphorylation (has00190) and ATP metabolic process (GO:0046034) pathways. In addition, the three inhibitory subtypes had higher scores for the above two pathways than did the other neuronal subtypes (Supplementary Figures S11A, B). These findings suggest that the three inhibitory subtypes may have a greater ability to generate ATP than the other subtypes.

A comparison of the epilepsy and control groups revealed that the epilepsy group had significantly higher scores for the oxidative phosphorylation and ATP biosynthesis pathways in most neuronal subgroups (Figures 5B, C; Supplementary Figures S11C, D). We also analyzed the score ratio between different neuronal subtypes and observed comparable score ratios for the oxidative phosphorylation signaling pathway in most neuronal subtypes (Figure 5D). However, the VIP_CRH subtype had the highest score ratio for the ATP metabolic process signaling pathway (Figure 5E).

### Alterations in FS/PV neurons in the brain tissues of FCD patients

**FIGURE 6:**
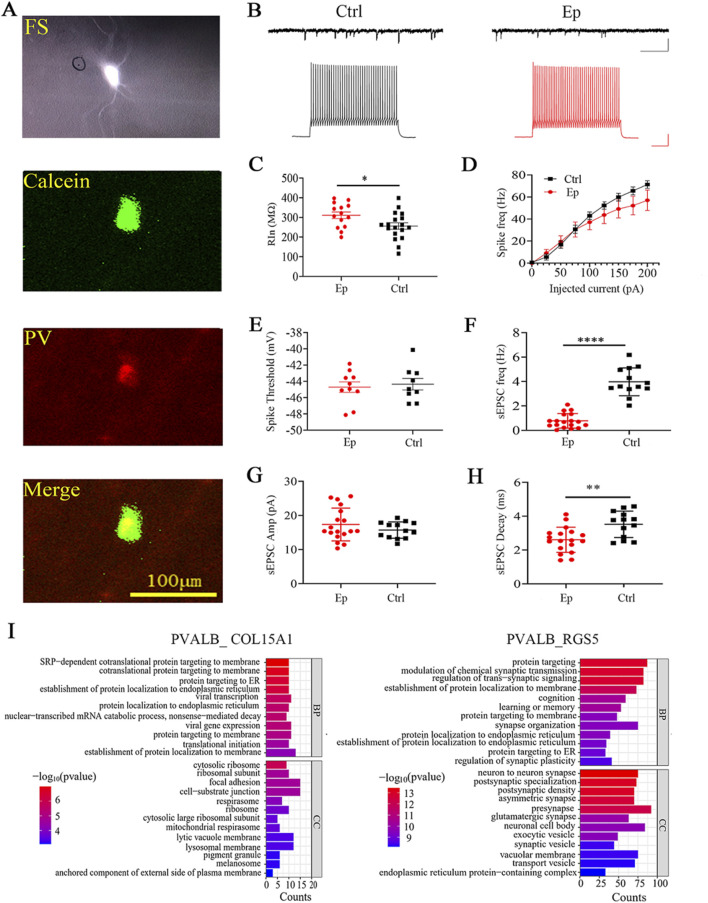
Alterations in the electrophysiological properties of FS neurons in brain tissue sections from pediatric FCD patients. **(A)** Representative images of recorded FS neurons and immunostaining with an anti-parvalbumin antibody after fixation. A green fluorescent dye (calcein) was included in the recording electrode, while red fluorescence indicates PV staining. Scale bar, 100 µm. **(B)** Sample traces of sEPSCs and spiking in FS neurons in the control and epilepsy groups. Scale bars, 50 pA/500 ms (sEPSCs) and 25 mV/100 ms (spikes). **(C)** Quantification of the input resistance (R_in_) of the recorded FS neurons in the epilepsy and control groups. **(D)** Spike frequencies following the injection of depolarizing currents in FS neurons in the epilepsy and control groups. **(E)** Spike threshold of FS neurons in the epilepsy and control groups. **(F)** Frequencies of sEPSCs in FS neurons in the epilepsy and control groups. **(G)** Amplitudes of sEPSCs in FS neurons in the epilepsy and control groups. **(H)** Decay time of sEPSCs in the epilepsy and control groups. **(I)** GO enrichment analysis of DEGs in the PVALB_COL15A1 and PVALB_RGS5 subtypes. *, p < 0.05; **, p < 0.01; ****, p < 0.0001.

The above results, including the significant epilepsy-related alterations in select neuronal subtypes, E/I balance-related pathways, and ATP production/energy metabolism, indicate that the PVALB_RGS5 subtype is a prominent inhibitory subtype. Parvalbumin (PV) neurons are the most widely studied neuronal subtype in epilepsy (Godoy et al., 2022), as their inhibition can suppress neuronal spiking and prevent seizure onset (Krook-Magnuson et al., 2013). A high percentage of PV neurons display high-frequency spiking (FS) in response to the injection of depolarizing current; hence, they can be identified via patch-clamp recording in acute sections from brain tissues that were obtained during surgery. We identified FS neurons in acute brain sections from the epilepsy and control groups. We performed HE staining to determine whether the recorded brain sections belonged to the control group or the epilepsy group (see Methods) (Supplementary Figure S12A). We included a green fluorescent dye in the internal recording solution, which diffused into the neurons during recording. The fixed sections were stained with PV-specific antibodies (red fluorescence). The overlap of green and red fluorescence within a single neuron confirmed that the recorded neuron was PV positive (Figure 6A). We found no difference in the intrinsic excitability of FS neurons between the epilepsy and control groups (Figures 6B, D). The input resistance was significantly greater (Figure 6C) and the sEPSC frequency and decay time were lower (Figures 6F, H) in the epilepsy group than in the control group, but there was no difference in the spike threshold (Figure 6E) or sEPSC amplitude (Figure 6G). These results indicate that certain intrinsic neuronal and synaptic properties are altered in FS/PV neurons in the brain tissues of FCD patients.

Since only one of the two PV-neuron subtypes exhibited prominent transcriptomic changes related to epilepsy, we examined the transcriptomic differences between these two PV subtypes in epilepsy. GO functional analysis revealed that the DEGs in the PVALB_COL15A1 subtype were enriched predominantly in pathways associated with proteins that target the membrane and ribosome (Figure 6I; Supplementary Figure S12E). On the other hand, the DEGs in the PVALB_RGS5 subtype were enriched in pathways related to glutamatergic synapses, asymmetric synapses, presynapses, GABAergic synapses, and synaptic transmission pathways (Figure 6I; Supplementary Figure S12F). Furthermore, KEGG analysis revealed that only the DEGs in the PVALB_RGS5 subgroup were enriched in the oxidative phosphorylation pathway. These transcriptomic differences indicate that only the PVALB_RGS5 subtype is related to epilepsy. Overall, the DEGs in the PVALB_RGS5 subtype and the electrophysiological results suggest altered excitatory synaptic transmission in PV neurons in the brain tissues of FCD patients.

### Alterations in astrocyte subtypes in the brain tissues of FCD patients

**FIGURE 7:**
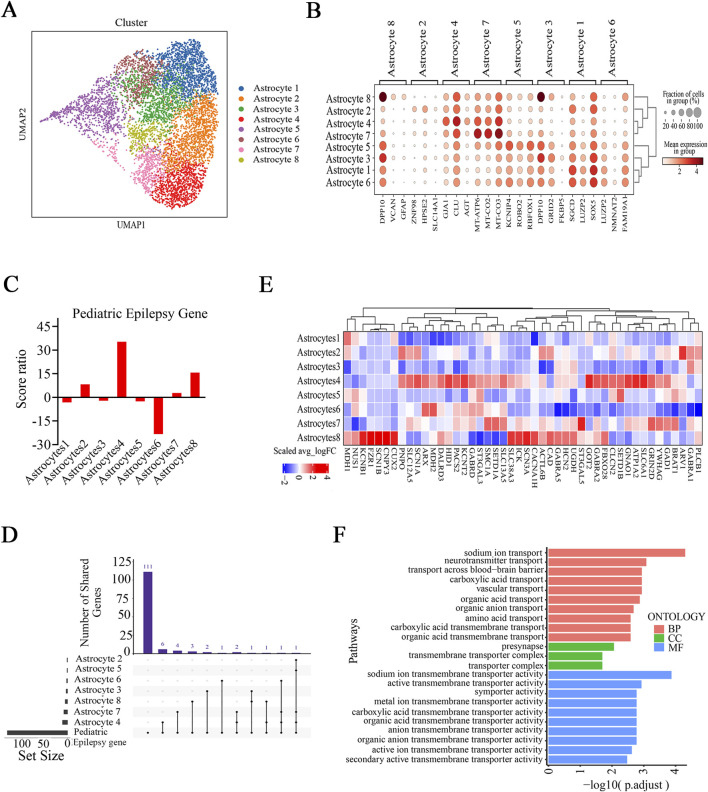
Selective astrocyte subtypes are related to disease in FCD patients. **(A)** UMAP embedding and cell type annotation for astrocyte subtypes. **(B)** Expression of known marker genes in all astrocyte subtypes. **(C)** Score ratios for the pediatric epilepsy gene set in astrocyte subtypes. **(D)** UpSet plot showing the numbers of overlapping pediatric epilepsy genes and DEGs (Ep vs. Ctrl) in identified astrocyte subtypes. **(E)** Relative expression (epilepsy (Ep) vs. control (Ctrl)) of pediatric epilepsy genes (from Zhang et al.) in astrocyte subtypes. **(F)** Enrichment analysis of overlapping pediatric epilepsy genes and DEGs (Ep vs. Ctrl) in astrocyte subtype 4. In **(D)**, the horizontal bar chart on the bottom left-hand side represents the entire size of each set. In the upper right-hand corner, the values on top of the bars indicate the numbers of shared genes at each intersection. Unconnected individual dots represent unique genes found in the astrocyte subtypes. Dots connected by lines indicate genes shared between two or more astrocyte subtypes, which correspond to the numbers on the bars in the upper section of the graph. In **(E)**, the rows represent astrocyte subtypes, and the columns represent epilepsy genes. The colors represent the scale (average log _2_ (fold change)). Fold change = G1/G2, where G1 and G2 represent gene expression in the epilepsy and control groups, respectively.

Our findings revealed potential alterations in neuronal energy metabolism within the epileptic zone (Figure 5). Considering the pivotal role of astrocytes in regulating cerebral energy and metabolism, we investigated whether substantial alterations in astrocytes occurred in the brain tissue of FCD patients. We classified astrocytes into eight subtypes, and there were no differences in the cell proportions between the epilepsy and control groups (Figures 7A, B; Supplementary Figure S13A). U-cell analysis of the pediatric epilepsy gene set revealed that only astrocyte subtype 4 presented a significantly higher score in the epilepsy group than in the control group and that astrocyte subtype 4 had a higher score ratio than the other subtypes (Figure 7C; Supplementary Figure S13B). In additionally, astrocyte subtype 8 had a higher score ratio than the other subtypes besides astrocyte subtype 4, although the magnitude of score ratio is smaller, suggesting a possible contribution of this subtype to epilepsy. A further examination of epilepsy-related gene sets confirmed that astrocyte subtype 4 presented significantly higher score ratios than did all other astrocyte subtypes (Supplementary Figures S13C, D). In addition, astrocyte subtype 6 presented a significantly greater score for the pediatric epilepsy gene set in the control group than in the epilepsy group, whereas there was no significant difference in scores for the epilepsy-related gene sets between the epilepsy and control groups (Supplementary Figures S13B–D). UpSet analysis of the pediatric epilepsy gene set revealed that among all subtypes, astrocyte subtype 4 exhibited the greatest number of intersecting pediatric epilepsy genes and DEGs (Figures 7D, E). The overlapping genes were *GAD1*, *SLC38A3*, *SLC13A5*, *GNAO1*, *SLC6A1*, *FBXO28*, *PACS2*, *ATP1A2*, *SCN1A*, and *YWHAG*, which were enriched in signaling pathways associated with transmembrane transport and transmembrane transporter activity (Figure 7F). Collectively, these results indicate that astrocyte subtype 4 has the strongest association with epilepsy; hence, it may play a role in the pathophysiological processes of epilepsy.

**FIGURE 8:**
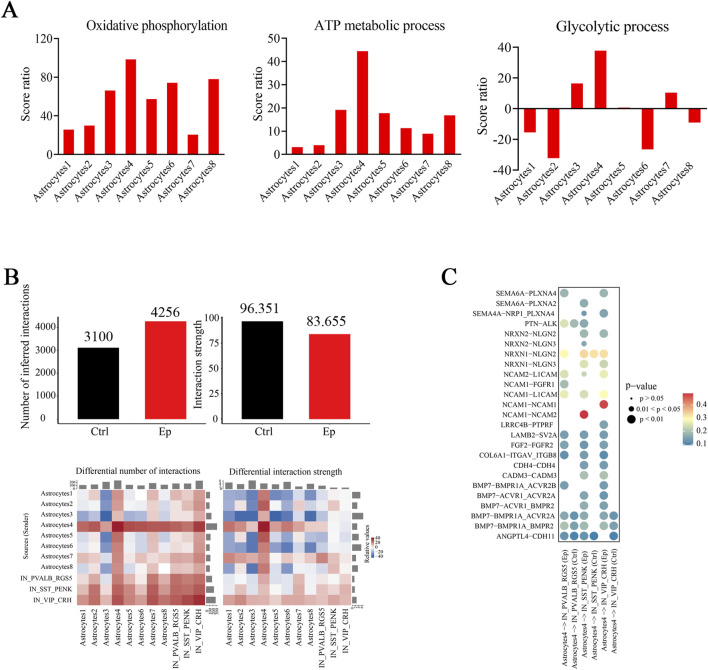
Mitochondrial functions and cell‒cell interactions are altered in select astrocyte subtypes in the brain tissues of pediatric FCD patients. **(A)** Score ratios for oxidative phosphorylation (left), the ATP metabolic process (middle), and the glycolytic process (right) in astrocyte subtypes. **(B)** The total number of interactions is shown on the left, and the strength of the interaction pairs in the epilepsy and control groups is shown on the right (upper). Below are the differences in the number and strength of interactions across different cell types in the epilepsy and control groups. **(C)** Interaction pairs between astrocyte subtype 4 and the three inhibitory neuronal subtypes were significantly different between the epilepsy and control groups. The colors represent the strengths of the interaction pairs.

We conducted U-cell analysis of two pivotal energy-related pathways: the ATP metabolic process (GO:0046034) and oxidative phosphorylation (GO:0006119). Most astrocyte subtypes exhibited higher oxidative phosphorylation scores in the epilepsy group than in the control group (Supplementary Figure S13E). In contrast, only three astrocyte subtypes presented significantly higher scores for the ATP metabolic process in the epilepsy group than in the control group (Supplementary Figure S13F). Notably, astrocyte subtype 4 had the highest score ratio, which suggests potentially increased ATP consumption in these cells (Figure 8A). Given that astrocytes primarily use glycolysis to supply energy to neurons, we analyzed scores for this pathway. Astrocyte subtype 4 was the only subtype with a significantly greater score ratio in the epilepsy group than in the control group, whereas astrocyte subtype 2 had a significantly greater score in the control group than in the epilepsy group, which suggests that these subtypes have distinct roles in epilepsy (Figure 8A; Supplementary Figure S13G). The DEGs in astrocyte subtype 4 were significantly enriched in pathways associated with synaptic function and energy metabolism (Supplementary Figure S13H).

Since select neuronal subtypes exhibited prominent associations in the brain tissues of FCD patients, we questioned whether interactions between these neuronal subtypes and astrocyte subtypes were enhanced. We observed a greater number of interaction pairs but a reduced interaction strength between these neuronal subtypes and astrocyte subtypes in the epilepsy group (Figure 8B; Supplementary Figure S14A). More interactions between astrocyte subtypes 4 and 7 and these three inhibitory neuronal subtypes occurred (Figures 8B; Supplementary Figures S14B, C). Further analysis of the interactions between astrocyte subtype 4 and the three inhibitory neuronal subtypes revealed some interaction pairs, which were related to the development, migration, synaptic formation, and plasticity of neurons, were selectively expressed in the epilepsy group (Figure 8C; Supplementary Figure S14D). A significantly greater strength of interactions between NCAM1-NCAM2 in the astrocyte subtype 4-SST_PENK and between NCAM1-NCAM1 in the astrocyte subtype 4-VIP_CRH in the epilepsy group were observed (Figure 8C). These findings suggest a specialized role for astrocyte subtype 4 in modulating the functional connectivity of these inhibitory neuronal subtypes, which may influence the pathophysiology of epilepsy.

## Discussion

By performing snRNA-seq of fresh brain tissues from pediatric FCD patients, we identified key neuronal and astrocyte subtypes that exhibited distinct molecular signatures and functional alterations, which indicated their contribution to epilepsy pathogenesis/pathology. The inhibitory neuron subtypes PVALB_RGS5, VIP_CRH, and SST_PENK and astrocyte subtype 4 are potential therapeutic targets for the more precise treatment of epilepsy. Our findings indicated that a few specific neuronal subtypes make prominent contributions to epilepsy; for example, the DEGs in PVALB_RGS5 were more strongly enriched in synaptic function-related pathways than the DEGs in the other subtypes. Consistently, we observed fewer excitatory synaptic inputs onto PV neurons in acute brain tissue sections from FCD patients, which is consistent with the results of a recent study (Cho et al., 2024).

The identification of specific epilepsy-related neuronal subtypes is an important finding of our study that corroborates previous findings in adult epilepsy patients and suggests the preferential involvement of certain subgroups in epilepsy (Pfisterer et al., 2020). The neuronal subtypes we identified are substantially different from those reported previously (Pfisterer et al., 2020), and this difference may have been caused by the type of epilepsy, patient age, or method of analysis used. For example, pediatric FCD, as a developmental disorder, is characterized by early seizure onset and severe drug resistance, which may lead to distinct transcriptomic alterations compared adult epilepsy (Pfisterer et al., 2020; Chung et al., 2023). Furthermore, the use of fresh tissue samples and single-nucleus RNA sequencing in our study allowed us to capture dynamic changes in gene expression that may not be detected in the studies using postmortem samples (Dachet et al., 2021; Heng et al., 2021). Postmortem tissues often exhibit a lower quantity of neuronal activity-dependent transcripts (Dachet et al., 2021), potentially reducing the probability to examine dynamic processes such as energy metabolism and synaptic function. In contrast, fresh brain tissue samples recapitulate a number of key features of refractory epilepsy (Morris et al., 2021), particularly in the genes associated with mitochondrial function and synaptic pathways.

The three inhibitory subtypes that we identified represent only one major subtype within each major subgroup of inhibitory neurons. Epilepsy may arise from functional alterations in neuronal synchrony that lead to hyperexcitability and subsequent seizure onset (Khazipov, 2016; Righes Marafiga et al., 2021). Although enhanced GABAergic inhibition generally counteracts seizures, increased inhibitory GABAergic signals have been reported to favor seizure initiation under certain conditions (Khazipov, 2016; Yekhlef et al., 2015). These complex and conflicting findings may be attributed to the existence of distinct subpopulations/subtypes in each inhibitory neuron subgroup, such as those expressing PV, SST, or VIP. Our results indicate that very few specific subtypes of PV, SST, and VIP neurons make predominant contributions to the pathology of epilepsy. This finding may have far-reaching implications for understanding the biological basis of epilepsy, improving epilepsy treatment, and identifying the biological properties and functions of each inhibitory neuronal subgroup.

Our study identified three key inhibitory neuronal subtypes, PVALB_RGS5, VIP_CRH, and SST_PENK. These subtypes exhibited distinct transcriptomic and functional alterations in pediatric FCD. Consistent with the reduced excitatory synaptic inputs in the epilepsy group revealed by our patch clamp recordings, the PVALB_RGS5 subtype showed significant enrichment in synaptic function-related pathways. Studies have shown that inhibiting VIP-neurons may enhance SST/PV-neuron outputs, and create a seizure choke point (Magloire et al., 2019; Paz and Huguenard, 2015). The VIP_CRH subtype showed the highest score ratio in the ATP metabolic process signaling pathway, indicating its potential role in regulating brain excitation and seizure initiation. Similarly, the SST_PENK subtype exhibited significant alterations in GABA receptor complex signaling pathways, particularly through the upregulation of *GABRB3*, a gene associated with severe developmental and epileptic encephalopathies (Absalom et al., 2022).

Patch clamp recording from fresh human brain tissue has provided us with important insights into the electrophysiological properties of the studied neurons. The reduced excitatory synaptic inputs observed in the FS/PV neurons aligns with the transcriptomic alterations, particularly in pathways related to synaptic function, in the PVALB_RGS5 subtype. The patch clamp results thus provide functional confirmation of the transcriptomic findings, highlights a critical role the PVALB_RGS5 subtype may play in modulating synaptic transmission in FCD-related epilepsy.

Interestingly, we observed a greater average percentage of mitochondrial genes in the three inhibitory subtypes. Mitochondrial dysfunction may be a contributor to seizure initiation or a consequence of excessively prolonged seizures (Singh et al., 2021; Puttachary et al., 2015; Zsurka and Kunz, 2015). During epilepsy, prolonged repetitive neuronal activity increases the demand for energy and disrupts the bioenergetic balance, which leads to an energy crisis and ATP shortage (Singh et al., 2021; Fei et al., 2020). Mitochondrial dysfunction may also cause a reduced energy supply and subsequent neuronal dysfunction. The higher average percentage of mitochondrial genes in the three inhibitory subtypes in control and epileptic brain tissues suggests that these subtypes may require more energy to maintain their normal functions. In support of the critical contribution of mitochondrial genes in these three inhibitory subtypes, higher scores for both the oxidative phosphorylation and ATP metabolic pathways were observed for these three inhibitory subtypes than for the other subtypes. Further research is needed to determine whether alterations in ATP metabolic pathways cause, result from, or play a compensatory role in epilepsy.

Astrocytes play important roles in supplying energy to neurons and are critical in the pathophysiology of epilepsy (Vezzani et al., 2022). We showed that astrocyte subtype 4 exhibited distinct metabolic characteristics and a reduced strength of interaction with neuronal subtypes. These findings point toward a possible shift in astrocyte‒neuron crosstalk that may favor epilepsy development/maintenance. However, we observed a greater interaction strength between astrocyte subtype 4 and the three inhibitory neuronal subtypes, which may play a critical role in modulating synaptic function and maintaining the E/I balance in the epileptic brain. Interestingly, astrocyte subtype 6 had higher scores in the control group than in the epilepsy group. These findings suggest that this subtype may play a protective or compensatory role in nonepileptic conditions, potentially contributing to the maintenance of normal brain function. As our tissue samples were not collected during seizures, our data reflect the profiles of resting astrocyte and neuronal subtypes.

We identified epilepsy-related genes whose expression was selectively and prominently altered in certain neuronal subtypes. Elevated expression of *GRIN2A* was observed in the PVALB_RGS5 subtype, and gain-of-function mutations in *GRIN2A* are linked to N-methyl-D-aspartate receptor overactivation, neuronal hyperexcitability (Chen et al., 2017) and severe developmental and epileptic encephalopathy phenotypes (Strehlow et al., 2019). In the VIP_CRH subtype, *GRIN2D* was selectively upregulated, and gain-of-function mutations in *GRIN2D* lead to significant increases in glutamate levels, glycine potency, and channel opening probability and a prolonged deactivation time (Li et al., 2016). We also identified novel epilepsy-associated genes. *CACNG7* expression was increased in the PVALB_RGS5 and SST_PENK subtypes, and *CACNG7* has been implicated in AMPA receptor trafficking and the modulation of receptor function (Kato et al., 2007). *CACNG8* expression was notably elevated in the SST_PENK subtype, and *CACNG8* has been identified as a target for protecting against seizures in animal models (Kato et al., 2016). Investigating the roles of these genes in different epilepsy subtypes may provide valuable insights into the mechanisms underlying epilepsy and potential therapeutic targets.

There are several reasons why only a handful of neuronal and astrocyte subtypes are strongly associated with epilepsy. First, higher energy demands in these subtypes may render them more vulnerable to an increase in activity or energy use in the brain. Alterations in E/I-related pathways in these epilepsy subtypes may lead to increased excitation in the brain, which in turn increases brain activity and energy demand. For example, the VIP_CRH subtype had the highest score ratio for the ATP metabolic signaling pathway, which suggests that this subtype has more powerful and effective control over excitation in the brain and the promotion of runaway excitation than other inhibitory neuron subtypes. Manipulating the VIP_CRH subtype may thus be more effective in preventing or controlling seizures than manipulating other subtypes. The unique functions of VIP neurons relative to other inhibitory subgroups position them as critically important players in the pathology of neurodevelopmental disorders, including epilepsy (Goff and Goldberg, 2021). Alternatively, epilepsy may occur as a consequence of impaired energy adaptation, particularly in inhibitory neurons. This impairment can lead to insufficient inhibition, thereby compromising the ability to effectively control seizures. Second, these three inhibitory subtypes are developmentally more vulnerable to alterations or perturbations in the environment, especially during protracted cortical development. Environmental perturbation may be particularly relevant for inhibitory neurons.

In summary, our study highlights the importance of specific neuronal and astrocyte subtypes in the pathophysiology of pediatric drug-resistant FCD. The distinct transcriptomic and functional alterations observed in PVALB_RGS5, VIP_CRH, and SST_PENK subtypes, as well as astrocyte subtype 4, suggest that epilepsy may arise from disruptions in specific subpopulations rather than uniform dysfunction across all cell types. The use of fresh tissue samples and snRNA-seq allowed us to capture dynamic changes in gene expression and cellular interactions that may be missed in postmortem studies. These findings provide a foundation for future research aimed at developing targeted therapies that restore the E/I balance and prevent seizure generation in patients with drug-resistant epilepsy. However, distinguishing whether the observed changes are etiologically linked to epilepsy or represent compensatory adaptations is challenging. In addition, our study focused primarily on neuronal and astrocytic subtypes because of their clear relevance to the pathophysiology of epilepsy. We did not conduct a detailed analysis of microglia. Given the emerging importance of microglia in epilepsy, future study is warranted to explore this topic. Addressing these challenges and developing methods to selectively study these specific neuronal subtypes may provide further insights into the disease mechanism and potentially to the development of new treatments for FCD.

## Data Availability

The raw sequencing data reported in this paper have been deposited in the China National Center for Bioinformation at https://ngdc.cncb.ac.cn/gsa-human, reference number GSA-Human: HRA007537.

## Supplementary material

SUPPLEMENTARY FIGURE S1 Representative HE-stained brain sections from epilepsy patients and control patients. **(A)** Representative HE-stained samples from the epilepsy group. **(B)** Representative HE-stained samples from the control group. Scale bar: 100 µm.

SUPPLEMENTARY FIGURE S2 Information on the epilepsy and control patient groups. **(A)** UMAP representation of excitatory and inhibitory neuronal subtypes in all samples. **(B)** UMAP representation of excitatory and inhibitory neuronal subtypes in the epilepsy and control groups. **(C)** Age of patients in the epilepsy and control groups. **(D)** Median gene numbers in the epilepsy and control groups. **(E)** Proportions of all excitatory neuronal subtypes in the epilepsy and control groups. **(F)** Proportions of all inhibitory neuronal subtypes in the epilepsy and control groups.

SUPPLEMENTARY FIGURE S3 Expression of epilepsy-related genes in neuronal subtypes. **(A)** UMAP representation of sores for epilepsy genes identified with DisGeNET (C0014544) in both excitatory and inhibitory neuronal subtypes in the epilepsy and control groups. Red represents high scores (top 25%), and gray represents low sores (0%–75%). **(B)** U scores of epilepsy genes identified with DisGeNET in excitatory and inhibitory neurons in the epilepsy and control groups. **(C)** U scores of pediatric epilepsy genes in excitatory and inhibitory neuronal subtypes in the epilepsy and control groups. **(D)** Score ratios of two epilepsy-related gene sets (from Zhang et al. and from Macnee et al.) in excitatory and inhibitory neuronal subtypes. *, p < 0.05; **, p < 0.01; ***, p < 0.001; ****, p < 0.0001.

SUPPLEMENTARY FIGURE S4 UpSet plot analysis of epilepsy-related gene set. **(A)** UpSet plot showing the numbers of overlapping epilepsy-related genes (from Zhang et al.) and DEGs (epilepsy vs Ctrl) in identified neuronal subtypes. **(B)** UpSet plot showing the numbers of overlapping epilepsy-related genes (from Macnee et al.) and DEGs (epilepsy vs Ctrl) in identified neuronal subtypes. The horizontal bar chart on the bottom left-hand side shows the entire size of each set. In the upper right-hand corner, the values positioned on top of the bars indicate the numbers of shared genes in each intersection. Unconnected individual dots represent unique genes found in the neuronal subtypes. Dots connected by lines indicate genes shared between two or more neuronal subtypes, corresponding to the numbers on the bars in the upper section of the graph.

SUPPLEMENTARY FIGURE S5 GO functional enrichment analysis of DEGs related to neuronal function-related pathways. The colors represent the –log_10_ values (*P* values).

SUPPLEMENTARY FIGURE S6 U scores of neurodevelopment-associated epilepsy genes in excitatory and inhibitory neuronal subtypes in the epilepsy and control groups. *, p < 0.05; **, p < 0.01; ***, p < 0.001; ****, p < 0.0001.

SUPPLEMENTARY FIGURE S7 UpSet plot analysis of glutamate receptor and GABA receptor related gene set. **(A)** UpSet plot showing the numbers of overlapping genes in glutamate receptor signaling pathway and DEGs (epilepsy vs Ctrl) in identified excitatory neuronal subtypes. **(B)** UpSet plot showing the number of overlapping genes in the GABA receptor complex signaling pathway and DEGs (epilepsy vs Ctrl) in the identified excitatory neuronal subtypes. The horizontal bar chart on the bottom left-hand side shows the entire size of each set. In the upper right-hand corner, the values on top of the bars indicate the numbers of shared genes in each intersection. Unconnected individual dots represent unique genes found in the neuronal subtypes. Dots connected by lines indicate genes shared between two or more neuronal subtypes, corresponding to the numbers on the bars in the upper section of the graph.

SUPPLEMENTARY FIGURE S8 U cell analysis of E/I-related signaling pathways and differences in cell‒cell interactions between the epilepsy and control groups. **(A)** U scores for the glutamate receptor signaling pathway (GO:0007215) in excitatory and inhibitory neuronal subtypes in the epilepsy and control groups. **(B)** U scores for the GABA receptor complex signaling pathway (GO:1902710) in excitatory and inhibitory neuronal subtypes in the epilepsy and control groups. **(C)** Score ratio for the glutamate receptor signaling pathway in excitatory and inhibitory neuronal subtypes. **(D)** Score ratio for the GABA receptor complex signaling pathway in excitatory and inhibitory neuronal subtypes. **(E)** The total number (left) and strength (right) of interaction pairs between the epilepsy and control groups. **(F)** Differences in the strength of interactions in individual neuronal subtypes between the epilepsy and control groups. *, p < 0.05; **, p < 0.01; ***, p < 0.001; ****, p < 0.0001.

SUPPLEMENTARY FIGURE S9 Average percentage of mitochondrial genes in neuronal subtypes in individuals from the epilepsy group. Owing to space limitations, the x-axis labels are consistent across all panels and are shown only in the last panel.

SUPPLEMENTARY FIGURE S10 Average percentage of mitochondrial genes in neuronal subtypes in individuals from the control group. Owing to space limitations, the x-axis labels are consistent across all panels and are shown only in the last panel.

SUPPLEMENTARY FIGURE S11 U cell analysis of oxidative phosphorylation and the ATP metabolic process in neuronal subtypes. **(A)** U scores for both the oxidative phosphorylation pathway and the ATP metabolic process in inhibitory neuronal subtypes. **(B)** U scores for both the oxidative phosphorylation pathway and the ATP metabolic process in excitatory neuronal subtypes. **(C)** U scores for the oxidative phosphorylation pathway in excitatory and inhibitory neuronal subtypes between the epilepsy and control groups. **(D)** U scores for the ATP metabolic process in excitatory and inhibitory neuronal subtypes between the epilepsy and control groups. *, p < 0.05; **, p < 0.01; ***, p < 0.001; ****, p < 0.0001.

SUPPLEMENTARY FIGURE S12 Electrophysiological properties of FS-neurons and the top 20 DEGs in both PV subtypes in the epilepsy and control groups. **(A)** Experimental procedure for patch clamp recording in brain slices from pediatric FCD patients. Scale bar: 100 µm. **(B)** AHP latency (left) and peak (right) in FS neurons from the epilepsy and control groups. **(C)** Quantification of spike amplitude in FS neurons from the epilepsy and control groups. **(D)** Quantification of peak-to-trough amplitudes in spikes of FS neurons in the epilepsy and control groups. **(E)** Quantification of the spike trough amplitude in FS neurons in the epilepsy and control groups. ***, p < 0.001. **(F)** Top 20 significantly up- or downregulated DEGs in the PVALB_COL15A1 subtype between the epilepsy and control groups. **(G)** Top 20 significantly up- or downregulated DEGs in the PVALB_RGS5 subtype between the epilepsy and control groups.

SUPPLEMENTARY FIGURE S13 Changes in the expression of genes in epilepsy-related gene sets and those involved in energy-related signaling pathways in select astrocyte subtypes. **(A)** Proportions of all astrocyte subtypes in the epilepsy and control groups. **(B)** U score for the pediatric epilepsy gene set in astrocyte subtypes between the epilepsy and control groups. **(C)** U score (left) and score ratio (right) for the epilepsy-related gene set (from Zhang et al.) in astrocyte subtypes. **(D)** U score (left) and score ratio (right) for the epilepsy-related gene set (from Macnee et al.) in astrocyte subtypes. **(E)** U score for the oxidative phosphorylation signaling pathway in astrocyte subtypes between the epilepsy and control groups. **(F)** U score for the ATP metabolic process in astrocyte subtypes between the epilepsy and control groups. **(G)** U score for the glycolytic process in astrocyte subtypes between the epilepsy and control groups. **(H)** GO and KEGG functional analyses of DEGs in astrocyte subtype 4. *, p < 0.05; **, p < 0.01; ***, p < 0.001; ****, p < 0.0001.

SUPPLEMENTARY FIGURE S14 Interactions between astrocytes and either other astrocyte subtypes or other neuronal subtypes. **(A)** The total number (left) and strength (right) of interaction pairs in the epilepsy and control groups. **(B)** Differences in the strength of interactions between individual astrocyte subtypes in the epilepsy and control groups. **(C)** Differences in the number and strength of interaction pairs among distinct cell subtypes in the epilepsy and control groups. **(D)** The interaction pairs between astrocyte subtype 4 and neuronal subtypes significantly differed between the epilepsy and control groups. The colors represent strength of the interaction pairs.
